# EphA2 super-enhancer promotes tumor progression by recruiting FOSL2 and TCF7L2 to activate the target gene EphA2

**DOI:** 10.1038/s41419-021-03538-6

**Published:** 2021-03-12

**Authors:** Shuang Cui, Qiong Wu, Ming Liu, Mu Su, ShiYou Liu, Lan Shao, Xiao Han, Hongjuan He

**Affiliations:** School of Life Science and Technology, State Key Laboratory of Urban Water Resource and Environment, Harbin Institute of Technology, Harbin, 150001 Heilongjiang China

**Keywords:** Cancer models, Cancer prevention

## Abstract

Super-enhancers or stretch enhancers (SEs) consist of large clusters of active transcription enhancers which promote the expression of critical genes that define cell identity during development and disease. However, the role of many super-enhancers in tumor cells remains unclear. This study aims to explore the function and mechanism of a new super-enhancer in various tumor cells. A new super-enhancer that exists in a variety of tumors named EphA2-Super-enhancer (EphA2-SE) was found using multiple databases and further identified. CRISPR/Cas9-mediated deletion of EphA2-SE results in the significant downregulation of its target gene *EphA2*. Mechanistically, we revealed that the core active region of EphA2-SE comprises E1 component enhancer, which recruits TCF7L2 and FOSL2 transcription factors to drive the expression of EphA2, induce cell proliferation and metastasis. Bioinformatics analysis of RNA-seq data and functional experiments in vitro illustrated that EphA2-SE deletion inhibited cell growth and metastasis by blocking PI3K/AKT and Wnt/β-catenin pathway in HeLa, HCT-116 and MCF-7 cells. Overexpression of EphA2 in EphA2-SE^−/−^ clones rescued the effect of EphA2-SE deletion on proliferation and metastasis. Subsequent xenograft animal model revealed that EphA2-SE deletion suppressed tumor proliferation and survival in vivo. Taken together, these findings demonstrate that EphA2-SE plays an oncogenic role and promotes tumor progression in various tumors by recruiting FOSL2 and TCF7L2 to drive the expression of oncogene EphA2.

## Introduction

Super-enhancers are larger clusters of transcriptional enhancers that play key roles in driving tumorigenesis^1^. Compared with typical enhancers, super-enhancers vary in size, transcription factors (TFs) density and content, and the ability to induce transcription^2^. They are distinguished by the enrichment of the MED1 signal. When the signal exceeds a certain threshold, the enhancer is considered to be a super-enhancer^2^. Super-enhancers are highly transcribed and occupied by numerous TFs, mediators, and enhancer epigenetic modification marks, such as histone H3 lysine 27 acetylation H3K27ac^3^.

Mounting evidence has demonstrated that super-enhancers play important roles in regulating cell identity, cell fate, stem cell pluripotency, and even tumorigenesis^1,2,4,5^. Super-enhancers likely promote the expression of their respective associated genes, such as *Sox2, Gata2*, and adipogenic-specific genes^6–8^. Previous data published in peer-reviewed journals reported that super-enhancers drive high levels of transcription of oncogenes in cancer cells^9,10^. In this study, we identified EphA2 (Eph type-A receptor 2)-associated super-enhancer EphA2-SE, which exists in five tumor cells in four super-enhancer databases. EphA2 is a member of the Eph (Erythropoietin-Producing Human Hepatocellular) receptor family, which includes an amino-terminal extracellular ligand-binding region, a transmembrane domain, and an intracellular enzyme domain^11^. Although EphA2 forms a receptor-ligand complex with ephrin 1–5, five different ligands, through the extracellular ligand-binding region, its main binding ligand is ephrinA1^12^. EphA2 activates cytoplasmic tyrosine phosphatase leading to auto-phosphorylation and tyrosine phosphorylation of a large number of downstream intracellular substrate protein molecules and initiating different signaling pathways to signal level transport^13^. In normal cells, EphA2 is degraded after phosphorylation; however, in tumor cells, EphA2 is dephosphorylated and found to be highly associated with the development of various types of cancer^11,14^, including cervical cancer^15^, ovarian cancer^16^, colon cancer^17^, breast cancer^18^, non-small-cell lung cancer^19^ and prostate cancer^20^. However, the mechanisms by which the EphA2-associated super-enhancer and its cofactors regulate its target genes are not clear, and their biological functions in cancer cells remain unexplored.

In the present study, we first identified a new super-enhancer EphA2-SE using the epigenetic modification H3K27ac and ChIP assay, and further characterized its constituent enhancers via the combination of luciferase reporter assays. Analysis of transcription factor occupancy showed that TCF7L2 and FOSL2 TFs bind to super-enhancer region and regulate *EphA2* expression. Importantly, deletion of EphA2-SE was shown to suppress cell proliferation, invasion, migration and tumor progression by blocking PI3K/Akt and WNT/β-catenin signaling pathway. Overexpression of EphA2 in EphA2-SE^−/−^ clones promotes cell proliferation and metastasis. These results highlight the important role of EphA2-SE in treating multiple tumors.

## Materials and methods

### Cell culture

HeLa, HCT-116, MCF-7, A549 and Panc-1 cells were cultured in DMEM (GIBCO, USA) containing 10% fetal bovine serum with penicillin/streptomycin in a humidified incubator.

### ChIP

ChIP assays were performed using a ChIP assay kit (UpstateBiotechnology, USA) following the manufacturer’s instructions. Cells were fixed with 1% formaldehyde at 37 °C for 10 min and then incubated with a final concentration of 0.125 M glycine for 5 min at room temperature to terminate the crosslinking. After washing with cold PBS, cells were lysed with SDS lysis buffer. The cross-linked DNA was sonicated to 200–1000 bp in length and then incubated with anti-H3K27ac antibody (#8173) or IgG (#3900) overnight at 4 °C (CST, USA). Immunecomplexes precipitated with protein A beads were washed and reverse cross-linked. Purified DNA was used as templates for PCR amplification. The normal rabbit IgG was used as control. ChIP primers are listed in Supplementary Table 1.

### CRISPR/Cas9-mediated deletion of EphA2-SE and E1-E3 enhancer

The single-guide RNAs (sgRNAs) for deleting EphA2-SE and E1-E3 component enhancers were designed by CRISPRscan (https://www.crisprscan.org) and CRISPRdirect (http://crispr.dbcls.jp). sgRNAs were annealed with NEBuffer2 in 95 °C 5 min, 70 °C 10 min. Annealed double-stranded DNA was inserted into the CRISPR/Cas9 PX458 vector using *Bbs*I and *Bsa*I (NEB, USA). Then the purified recombinant plasmid was transfected into the HeLa, HCT-116, and MCF-7 cells in 24-well plates. The puromycin was added into cells after 48 h of transfection. After puromycin screening, cells were separated into 96-well plates by limiting dilution. PCR was amplified from the DNA isolated from homozygous (SE^−/−^) clones using external and internal primers of EphA2-SE. The knockout plasmids of E1-E3 enhancer were transfected for 48 h and added with appropriate puro for 72 h, then the cells were collected to test the knockout efficiency and gene expression. The PX458 vector has been modified, and the modification diagram is in the Supplementary Materials. Primers sequences can be found in Supplementary Table 1.

### RNA extraction and qRT-PCR

Total RNA was extracted from cells using RNAiso Plus (TaKaRa, Dalian, China) according to the manufacturer’s protocol, and then cDNA was synthesized by reverse transcription using PrimeScriptTM RT reagent Kit with gDNA Eraser (TaKaRa). qRT-PCR was performed by using FastStart Universal SYBR Green Master (Roche, Basel, Switzerland) on an ABI 7500 Real-Time PCR Systems. Results were analyzed using the relative quantitative method and mRNA expression of genes was normalized with *GAPDH*. Primers of qRT-PCR were shown in Supplementary Table 1.

### Western blotting analysis

Cells were lysed using RIPA lysis buffer with PMSF and Protease Inhibitor Cocktail (APEXBIO, USA), and the total protein content was determined by BCA method. Overall, 30 μg of protein was separated by 10% SDS-PAGE, and then transferred to PVDF membrane. After blocking with 5%/TBS skim milk, the membrane was incubated with specific primary antibody at 4 °C overnight. The blot was then washed and probed with a peroxidase-conjugated secondary antibody, and the signal was visualized using an ECL chromogenic kit (Tanon, Shanghai, China) with the Mini-REPORT Tetra Electrophoresis System (Bio-Rad, USA). The primary antibodies anti-Tubulin (#2146), EphA2 (#6997), FOSL2 (#19967), TCF7L2 (#2565), AKT (#2920), p-AKT (#15116), cyclin-E1 (#4129), GAPDH (#5174) (CST) and β-catenin (#51067-2-AP) (Proteintech) were diluted 1:1000 in TBST. The GV230-EphA2 overexpression vector was purchased from GeneChem (GeneChem, China). The blank GV230 vector was constructed by deleting EphA2 gene from the GV230-EphA2 vector.

### RNA-seq analysis

Three independent total RNAs were extracted from each cell line and then mixed well. The quality and purity of RNAs were checked using RNA electrophoresis and Nanodrop, respectively. Total RNA was provided to Personal Bio Company for transcriptome sequencing. The library was prepared using the Illumina mRNA-Seq sample preparation kit. After the library is constructed, the library is subjected to Paired-End sequencing based on the Illumina Hiseq sequencing platform. Differentially expressed genes (DEGs) were identified by absolute fold change >1.2 or <0.83. DAVID software^21,22^ was used to annotate GO functions of different genes, including cell components, biological processes and molecular functions.

### Luciferase reporter assays

The luciferase backbone vector is modified PGL4.10 (Promega, USA), and we inserted the TK promoter upstream of luciferase. The constituent enhancer regions and fragments were cloned into the upstream of TK promoter using *Kpn*I and *Xho*I, respectively. HeLa, HCT-116 and MCF-7 cells were seeded in 24-well plates, and then 200 ng of the constructed luciferase vector and 8 ng Renilla plasmids were co-transfected using lipofectamine 3000 (Invitrogen, USA). 40 pmol siFOSL2 or siTCF7L2, 200 ng constructed E1 luciferase vector and 8 ng Renilla plasmid were co-transfected into cells. After 48 h of transfection, cells were harvested and assayed by Dual-Luciferase Reporter Assay System (Promega, USA). The ratio of relative luciferase activity was normalized using Renilla luciferase activity. The deleted FOSL2 and TCF7L2 motif sequences in the E1 enhancer and amplification primers for each region were listed in Supplementary Table 1.

### siRNA

siRNAs against TCF7L2 and FOSL2 were designed using siDirect version 2.0 and DSIR. siRNAs were purchased from Gene Pharma (Gene Pharma, Suzhou, China). Cells in 12-well plates were transfected with lipofectamine 3000 (Invitrogen) at a final concentration of 40nM siRNA. After incubation for 48–72 h, cells were harvested for mRNA and protein analysis. The sequences of siTCF7L2 and siFOSL2 were listed in Supplementary Table 1.

### Proliferation and colony formation

Cells were seeded in 96-well plates and the procedure was performed as described previously^23^. The absorbance at 490 nm was measured in a microplate reader (Bio-Rad, USA) every 24 h at the indicated time. For colony formation, cells were seeded in six-well plates at an appropriate density. The colonies were fixed and stained according to the methods described previously^24^ after incubation for 10–15 days.

### Wound healing, cell invasion and migration

HeLa, HCT-116 and MCF-7 cells were cultured to confluent monolayer cells and scraped with a 200-μL micropipette tip. Cells were then incubated with serum-free medium to close the wound for 48 h.

For cell invasion and migration, 2 × 10^6^ HeLa, 1 × 10^6^ HCT-116 and 5 × 10^5^ MCF-7 cells were plated in the upper chamber of a Transwell chambers (Corning, USA) coated with or without Matrigel (BD Biosciences, USA). After incubation for 48 h, the cells were fixed in 4% paraformaldehyde for 30 min, then washed twice with PBS for 5 min, and stained with 0.1% crystal violet for 15 min. Five random fields were counted for each membrane under the microscope. Each assay was performed in triplicate.

### In vivo tumorigenesis assay

Nude mice were purchased from Charles Riverand and maintained according to institutional guidelines. A total of 5 × 10^6^ cells (HeLa and EphA2-SE-deficient cells) were injected into the dorsal flanks of 6-week-old nude mice, respectively. At least five mice were injected and observed for tumor formation for 4 weeks. The tumor volume was measured every 6 days from the 11th day until the mice were sacrificed. Five mice were randomly selected to calculate the volume according to the following formula: V = (length × width^2^)/2. Animal experiments were carried out in strict accordance with the Guide for the Care and Use of Laboratory Animals from the Harbin Institute of Technology (HIT) and approved by the Institutional Animal Care and Use Committee (IACUC) or Animal Experimental Ethics Committee of HIT.

### Statistical analysis

For statistical analysis, each experiment was performed at least three independent replicates. Statistical significance was tested by the two-tailed Student’s *t* test, with mean ± SD *p < 0.05, **p < 0.01 and ***p < 0.001 indicating statistically significant differences. Statistical analysis was produced by GraphPad Prism software.

### Data analysis

**Fig. 1:**
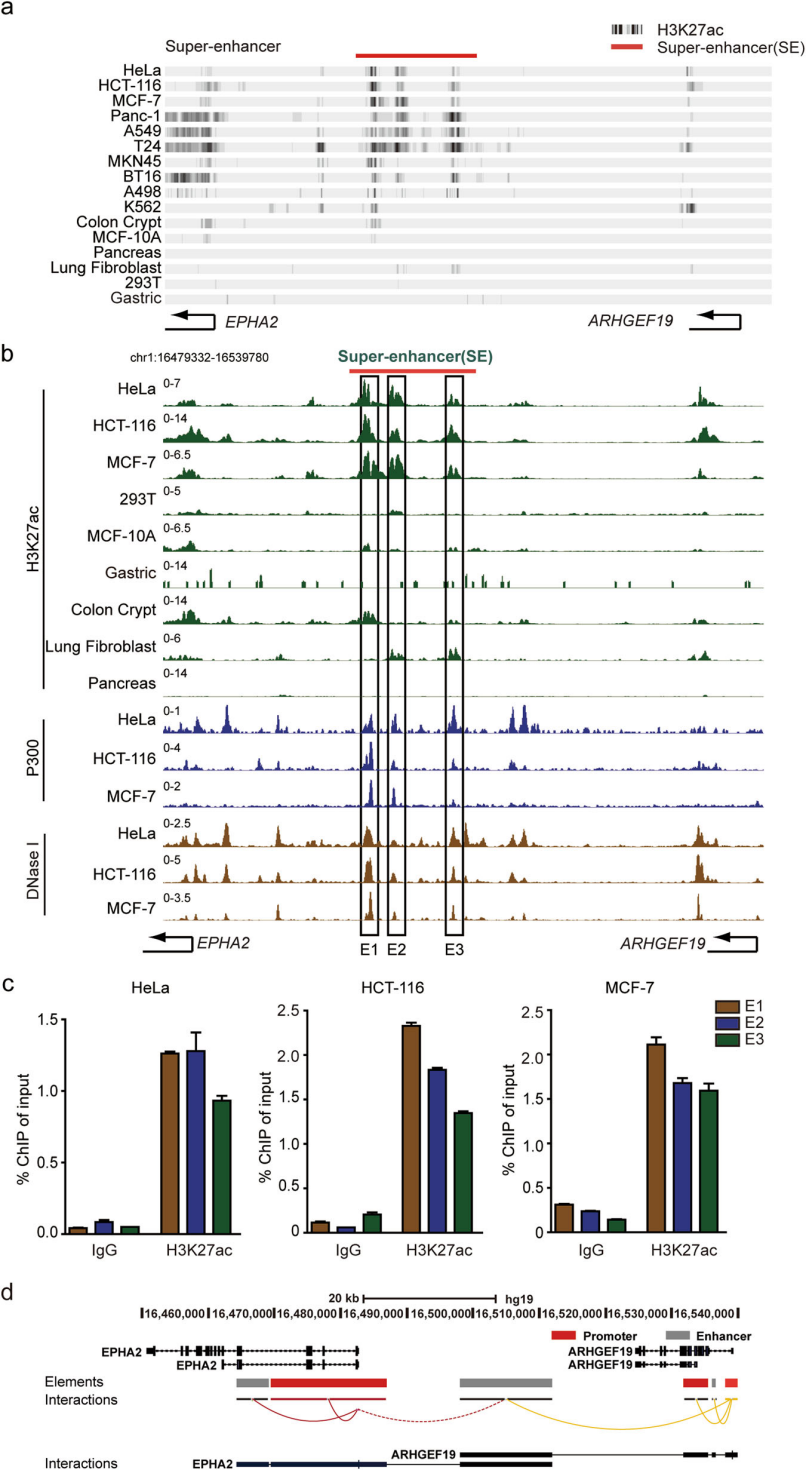
Identification of a super-enhancer in ten tumor cells. **a** ChIP-seq binding profiles for H3K27ac enrichment were used to identify the super-enhancer in a variety of cancer cells. **b** The constituent enhancers of the super-enhancer (E1-E3) were identified based on markers of H3K27ac, P300, and DNase I in HeLa, HCT-116 and MCF-7 cells. **c** ChIP-qPCR analysis of H3K27ac in E1-E3 component enhancers in HeLa, HCT-116 and MCF-7 cells. **d** Promoters interacting with the super-enhancer were analyzed by GeneHancer database.

Select ChIP-seq data in Cistrome Data Browser and visualize it in UCSC browser. For Fig. 1a, the track is displayed in the form of “Display mode = dense”. For Fig. 1b and Supplementary Fig. S1a, the track is displayed in the form of “Display mode = full”. The GEO accession IDs of all analyzed data sets are listed in Supplementary Table 2.

### Motif analysis

To find the binding motifs of TCF7L2 and FOSL2 on the E1 enhancer, we input the E1 enhancer sequence into JASPAR in FASTA format, and selected TFs for screening. Among the many results, we selected the top two sequences based on the score for verification.

### Gene ontology analysis

For gene ontology (GO) analysis, the R package cluster Profile was respectively used to analyze for the differential genes of the three cell lines.

### Accession numbers

RNA-seq sequencing data have been deposited in GEO under accession number GSE159353.

## Results

### Identification of a super-enhancer in multiple cell lines

Super-enhancers, which are characterized by H3K27ac enrichment, promote the expression of cell identity-related genes. Following previous work, classes of super-enhancers were found in a wide variety of tumors or cancer cells, such as KLF6-SE^25,26^ and MYC-SE^27–30^. Recently, there are four most commonly developed used databases for super-enhancers, including SEanalysis^31^, SEdb^32^, SEA^33^and dbSUPER^34^ databases. These databases use the ranking method based on the H3K27ac signal (ROSE) to summarize and classify SE regions of various tissues and cell types. In this work, a super-enhancer was found in A549, HeLa, MCF-7, HCT-116, Panc-1, T24, MKN45, BT16, A498 and K562 cancer cells by searching multiple databases. Through the public H3K27ac ChIP-seq data of the Cistrome Data Browser (http://cistrome.org/db/#/), we obtained the H3K27ac enrichment level of the super-enhancer in ten tumor cells. In addition to the histone modifications of ten types of tumor cells, six types of normal cells or tissues are also displayed, including normal tissues or cells of intestine, breast, pancreas, lung, kidney and stomach. Some of them are not enriched in H3K27ac modifications, such as pancreas and kidney tissues; some are enriched, but much lower than their corresponding cancer cells, such as intestine, breast, lung and stomach (Fig. 1a). These data indicate that the presence of super-enhancer is enriched in cancer cell lines, but generally absent in normal cell lines. In addition, to further investigate the potential super-enhancer, we analyzed this region of DNA based on enhancer-associated features. The results validated that this region exhibits higher H3K27ac enrichment as well as P300, DNase I hypersensitivity. Accordingly, we further divided this super-enhancer into three constituent enhancer regions, E1-E3 (Fig. 1b and Fig. S1a). ChIP experiments were performed to detect active histone modifications of component enhancers of the super-enhancer using the H3K27ac antibody. The results indicated that H3K27ac was enriched on each enhancer cluster, E1-E3, in HeLa, MCF-7, HCT-116, A549 and Panc-1 cells (Fig. 1c and Fig. S1b).

GeneHancer is a database comprising human regulatory elements (enhancers and promoters) and their inferred target genes^35^. The 4DGenome database obtains chromatin interactions through two methods: literature search and software prediction. The database currently covers low-throughput and high-throughput detection^36^. According to the two databases, the identified new super-enhancer is considered to interact with the promoters of two different genes, EphA2 and ARHGEF19. Therefore, these genes may be considered as the candidate target genes of this super-enhancer (Fig. 1d and Fig. S1c).

### CRISPR/Cas9-mediated EphA2-SE deletion significantly reduced *EphA2* expression and RNA-seq analysis

**Fig. 2:**
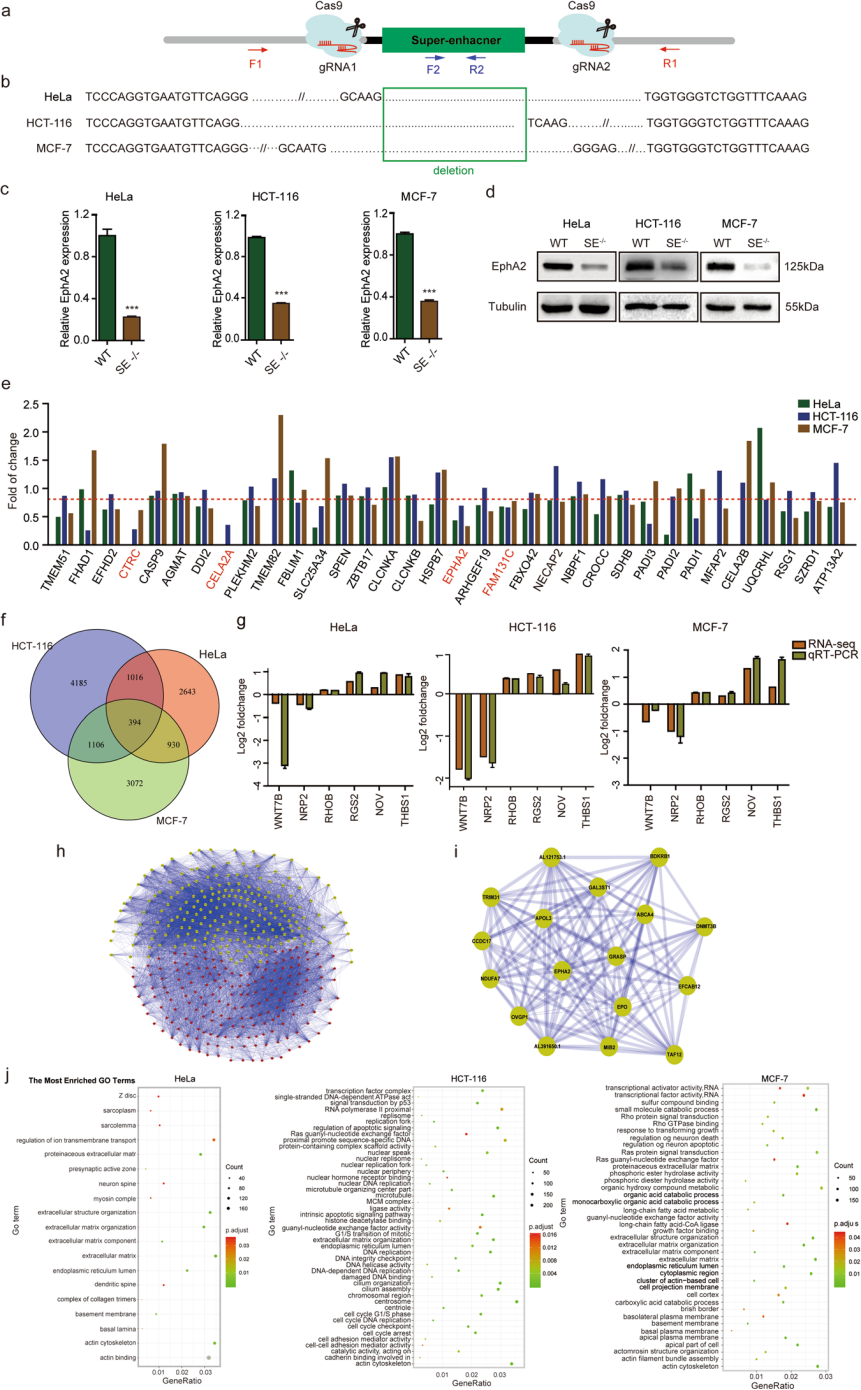
CRISPR/Cas9-mediated deletion of EphA2-SE and RNA-seq of EphA2-SE. **a** Schematic of CRISPR/Cas9-mediated deletion of the super-enhancer. **b** The deletion clones of the super-enhancer were identified through sequencing results. **c** The expression level of EphA2 in EphA2-SE-knockout cells was significantly decreased by qRT-PCR and **d** western blotting. Relative expression level was normalized by GAPDH. Error bars, mean ± SD. *n* = 3. *p* values were calculated using *t*-test. ***p < 0.001. **e** RNA-seq analyzes the gene expression of EphA2-SE within ~1 Mb. **f** Venn diagram of up-regulated genes and downregulated genes in HeLa, HCT-116 and MCF-7 cells (fold change >1.2 or <0.83). **g** Validation of RNA-seq analysis in EphA2-SE (SE^−/−^) clones. **h** Co-expression network map of 394 public differentially expressed genes. **i** A module with EphA2 as the core in co-expression network. **j** GO enrichment analysis of differentially expressed genes.

To investigate the target gene and functional role of the super-enhancer in cancer cells, the super-enhancer was specifically knocked out using the CRISPR/Cas9 system. Two independent sgRNAs targeting the full length of the super-enhancer were inserted into the CRISPR/Cas9 vector PX458 and then transfected into HeLa, HCT-116 and MCF-7 cells, respectively (Fig. 2a). External (F1/R1) and internal (F2/R2) primers for the super-enhancer region were further used to amplify genomic DNA isolated from cells (Fig. S2a). By sequencing, homozygous super-enhancer deletion clones (SE^−^^/−^) were obtained from HeLa, HCT-116 and MCF-7 cells, respectively (Fig. 2b). qRT-PCR and western blotting analysis showed that the expression of EphA2 was strongly downregulated in SE-knockout group (SE^−/−^) compared to that of the wild type in HeLa, HCT-116 and MCF-7 cells (Fig. 2c, d), however, the expression of ARHGEF19 was only altered in MCF-7 cells (Fig. S2b). Therefore, EphA2 is the target gene of this super-enhancer in HeLa, MCF-7 and HCT-116, while ARHGEF19 is only regulated in MCF-7 cells. In this study, we focused on the EphA2 that is significantly downregulated in all three cells, so the new identified super-enhancer was named as EphA2-SE. Taken together, the findings suggest that EphA2 is the target gene of EphA2-SE in the three cell types. In other words, EphA2-SE is a master super-enhancer that activates EphA2 expression in HeLa, HCT-116 and MCF-7 cells.

To further analyze the molecular mechanism underlying EphA2-SE regulation, RNA-seq analysis of HeLa, HCT-116 and MCF-7 homozygous cells was performed. The expression profile of all genes within ~1 Mb distance from EphA2-SE was analyzed from the RNA-seq. From Fig. 2c, it can be found that there are only four genes downregulated in the three cell lines of HeLa, HCT-116 and MCF-7, namely CTRC, CELA2A, EphA2 and FAM131C (Fig. 2e). From Fig. S2c, we can find that the expression of EphA2 is much higher than the other three genes. CTRC and CELA2A are almost not expressed. Studies have shown that the expression level of genes regulated by super-enhancers is much higher than those regulated by ordinary enhancers. Therefore, EphA2 is more likely to be regulated by the super-enhancer.

Overall, 4983, 6701 and 5502 DEGs were found in three cells, respectively (Fig. S2d). A total of 394 common DEGs with the same direction were obtained, and EphA2 expression was found to be significantly decreased in the three cancer cell lines (Fig. 2f). Of the 394 genes, many were shown to be related to tumor growth and metastasis, such as *THBS1*, *NOV*, *RGS2*, *WNT7B, RHOB* and *NRP2* (Fig. 2g). Given the similarities in gene expression, the possible interactions of gene products were analyzed to understand the interaction between genes and to identify core genes. The common differentially expressed 394 genes were mapped to a co-expression network (Fig. 2h). The MCOD cytoscape^37^ was used to mine 16 modules with more than five nodes, one of which contained 17 downregulated genes, and *EphA2* was the core gene (Fig. 2i). Moreover, functional enrichment analysis of DEGs in each cell revealed that in HCT-116 cell, DEGs were enriched in DNA replication and intercellular adhesion; in HeLa cell, DEGs were enriched in the cytoskeleton; in MCF-7 cell, DEGs were enriched in transcriptional activator activity and growth factor binding (Fig. 2j). Taken together, functional enrichment analysis of DEGs in each cell revealed that EphA2-SE plays a role in the development of tumor cells.

### Enhancer activity of EphA2-SE constituents and signaling modules at super-enhancer

**Fig. 3:**
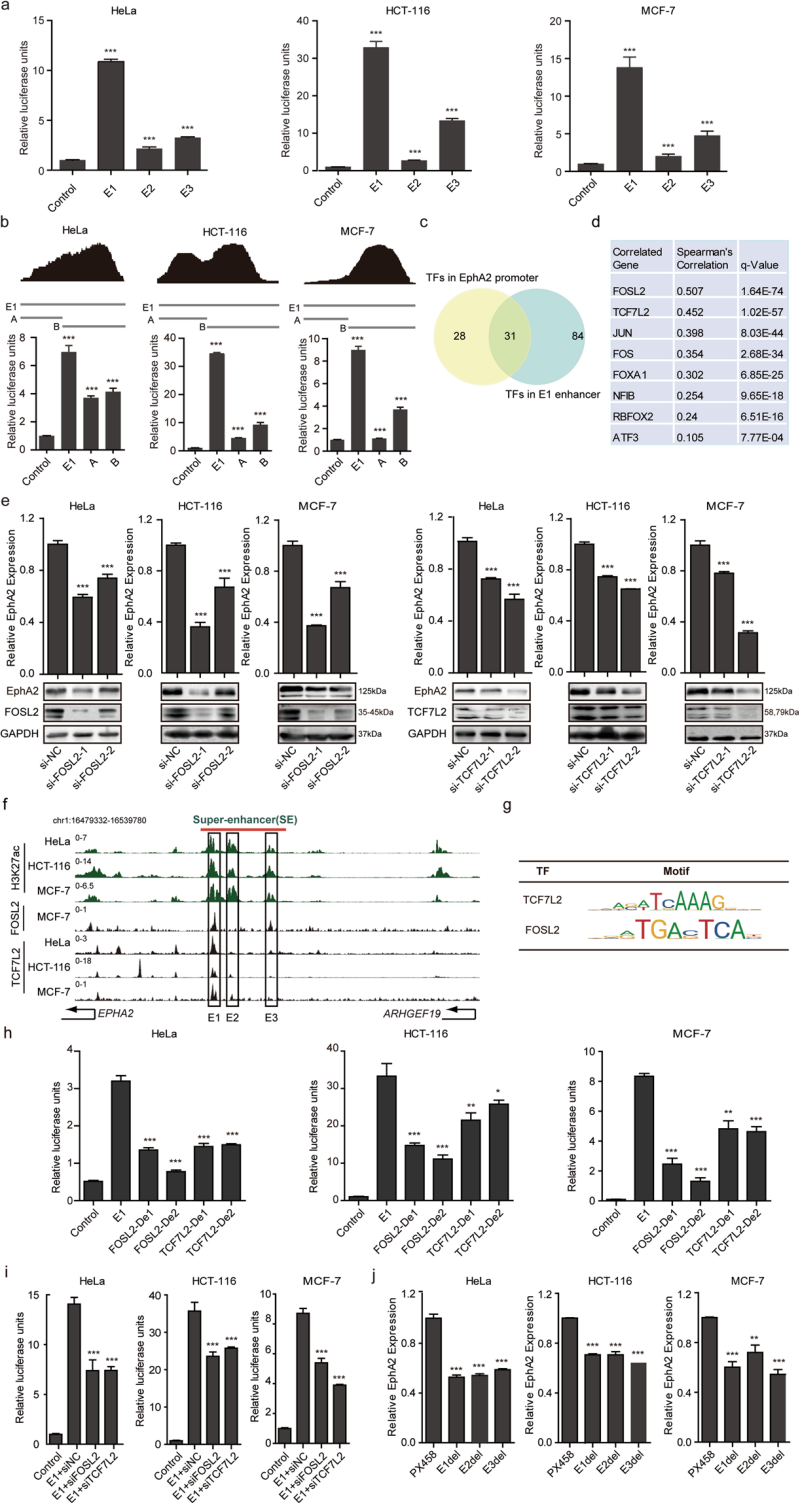
Activities of EphA2-SE constituents and transcription factors that bind to the E1 enhancer. **a** The enhancer activity of E1-E3 within the EphA2-SE region was measured by dual-luciferase reporter assays in HeLa, HCT-116 and MCF-7 cells, respectively. **b** Top, transcription factor density. The abscissa represents the E1 component enhancer region, and the ordinate represents the number of transcription factors. Bottom, the enhancer activity of the two fragments of E1 was measured. **c** Veen displays transcription factors that bind to both the E1 component enhancer and the EphA2 core promoter region. **d** Correlation analysis of 31 transcription factors bound in the E1 enhancer and core promoter regions. We use the Cbioportal website (https://www.cbioportal.org/) to retrieve data from the cell lines (Broad 2019, Novartis/Broad Nature 2012 and NCI Cancer Res 2012) databases. **e** The expression of EphA2 was detected by qRT-PCR and western blotting after knocking down FOSL2 and TCF7L2. **f** ChIP-seq profiles of FOSL2 and TCF7L2 transcription factors in E1-E3 component enhancers. **g** The motifs of FOSL2 and TCF7L2. **h** E1 enhancer activity decreases after the binding sites of FOSL2 and TCF7L2 are deleted. **i** The E1 enhancer activity was reduced after adding siFOSL2 and siTCF7L2, respectively. **j** The expression of EphA2 after partial deletion of E1-E3 component enhancers. Error bars, mean ± SD. *n* = 3. *p* values were calculated using *t*-test. *p < 0.05, **p < 0.01, ***p < 0.001.

The individual constituent enhancers E1-E3 of EphA2-SE were cloned into a luciferase reporter vector and found to exhibit higher activity compared with that in the control. The E1 constituent enhancer produced the largest signal in HeLa, HCT-116 and MCF-7 cells, followed by the E3 and E2 enhancers (Fig. 3a). Furthermore, according to the ChIP-seq database and the analysis of transcription factor enrichment density, the E1 enhancer was further divided into two ~500 bp fragments, namely E1-A and B (Fig. 3b, upper). The results revealed that the activity of each segment is lower than the entire E1 constituent. In addition, segment B showed more activity than the segment A, which is consistent with results for transcription factor enrichment density (Fig. 3b, bottom). These findings indicate that E1 constituent enhancer is the main active unit in the EphA2-SE.

We further explored the molecular mechanism of how EphA2-SE specifically regulates EphA2. Compared with typical enhancers, super-enhancers are occupied by high levels of TFs^1,2^ and stimulate the transcription of target genes more strongly. Therefore, the identification of key TFs is of great significance to the research of super-enhancers. According to the method of Deng et al.^38^ to identify TFs, we analyzed E1 component enhancer region and EphA2 core promoter region that bind to TFs and enriched with H3K27ac, H3K4me1 and H3K4me3 from ChIP-seq of ENCODE project. Among the 340 TFs of ENCODE project, we found that 115 TFs bind to the E1 component enhancer region, which overlaps with the region characterized by the enrichment of H3K27ac and H3K4me1 markers (Fig. S3a). Overall, 59 TFs bind to the core promoter region of EphA2, overlapping with the region characterized by the enrichment of H3K27ac and H3K4me3 markers (Fig. S3b). There are 31 overlapping TFs (Fig. 3c). Subsequently, the correlation between EphA2 and these 31 TFs was analyzed using the mRNA expression database of tumor cells in the TCGA database. The results showed that EphA2 has the strongest correlation with the two TFs FOSL2 (FOS-like antigen 2) and TCF7L2 (Transcription factor 7-like 2) (Fig. 3d). Next, the expression of FOSL2 and TCF7L2 was downregulated by siRNA and the expression level of the target gene EphA2 was greatly decreased, in terms of both mRNA and protein levels in all three cell lines (Fig. 3e). Meanwhile, HCT-116 and MCF-7 cells show the highest levels of enrichment of the TCF7L2 transcription factor on the E1 component enhancer and MCF-7 cell is rich in high concentration of FOSL2 in the E1 enhancer region. (Fig. 3f). JASPAR database contains the motif information of TFs (Fig. 3g), which can be used to predict the binding region of TFs and sequences. In the database, the binding sites on the E1 component enhancer were analyzed based on the motifs of TCF7L2 and FOSL2 TFs. According to the research method of Zhang’s et al.^39^, when the two binding sites of FOSL2 or TCF7L2 in the E1 component enhancer were deleted, the enhancer activity was greatly reduced (Fig. 3h). siRNA-mediated knockdown of FOSL2 and TCF7L2 leads to a significant decrease in E1 enhancer activity as compared to control siRNAs (Fig. 3i). These results indicate that the intact E1 enhancer was responsible for the high level of EphA2-SE activity and EphA2-SE regulated EphA2 expression by recruiting TCF7L2 and FOSL2 TFs to the E1 enhancer.

In order to simulate the chromatin structure inside the cell, we partially deleted the three component enhancers in HeLa, HCT-116 and MCF-7 cells. The three enhancer components E1-E3 knockout plasmids were transfected into three cells for 48 h, respectively. Then an appropriate concentration of puro was added for screening 72 h. Cells were collected to detect knockout efficiency and EphA2 expression. The results showed that the expression of EphA2 was significantly downregulated after the partial deletion of the E1-E3 component enhancers, but there was little difference between the different components (Fig. 3j and Fig. S3c).

### EphA2-SE deletion suppressed cell proliferation, migration, and invasion by decreasing the expression of EphA2 in vitro

**Fig. 4:**
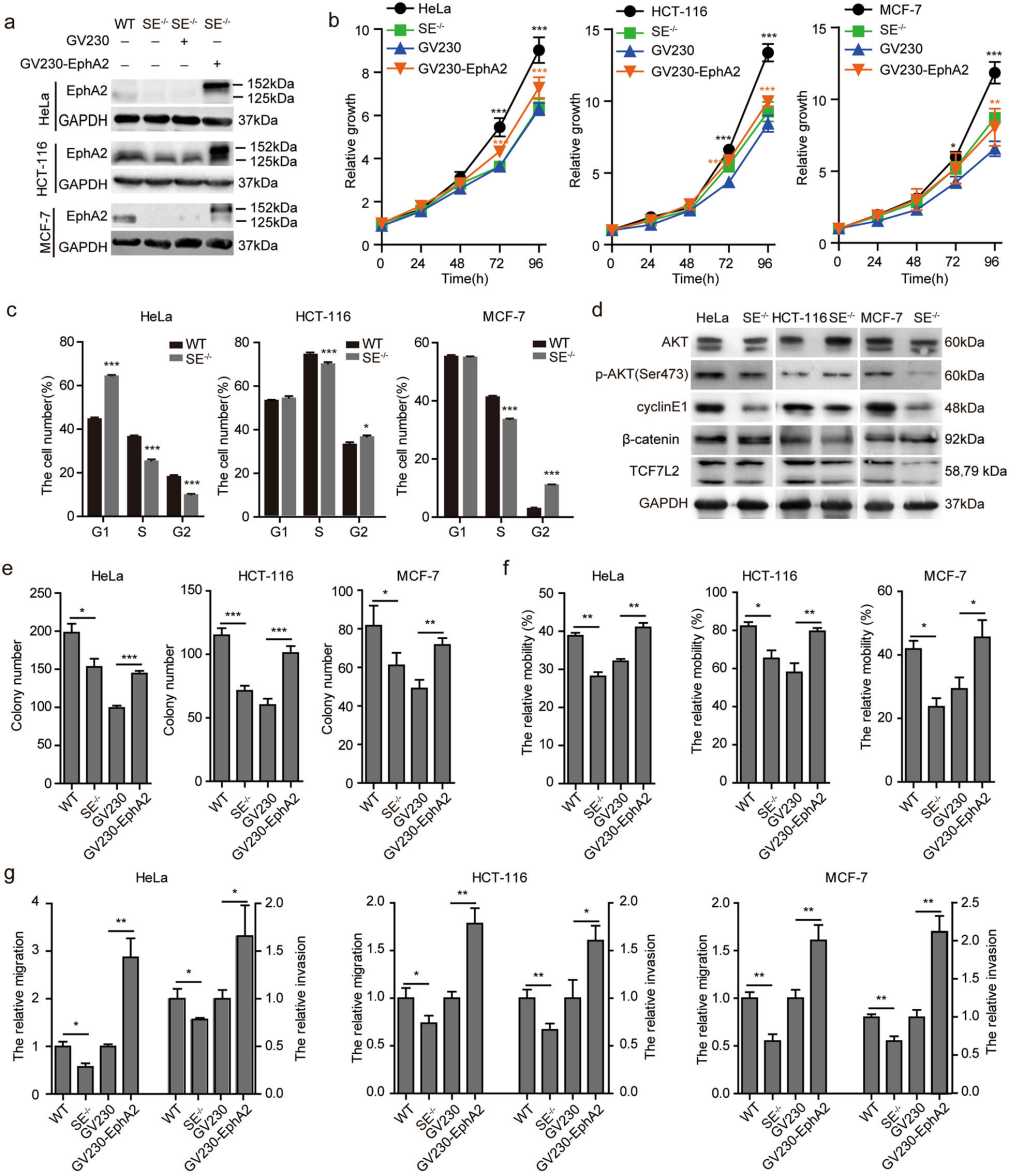
EphA2-SE knockout inhibited proliferation and migration of HeLa, HCT-116, and MCF-7 cells. **a** Expression level of EphA2 as measured by western blotting in Hela, HCT-116 and MCF-7 cells. The overexpression vector GV230-EphA2 was constructed to express EphA2-EGFP fusion protein, and then transfected into the EphA2-SE homozygous deletion SE^−/−^ cell lines. **b** Cell proliferation was measured using MTT assay after EphA2-SE knockout and EphA2 overexpression. **c** Flow cytometry was used to detect the cell cycle of HeLa, HCT-116 and MCF-7 cells. **d** Effect of EphA2-SE deletion on PI3K-AKT signaling pathway. **e** The summary graphs of the number of colonies counted. **f** The migration distance was calculated. **g** Migration and invasion efficiency measured by transwell assay. Error bars, mean ± SD. *n* ≥ 3. *p* values were calculated using *t*-test. *p < 0.05, **p < 0.01, ***p < 0.001. WT: wild type cell; SE^−/−^: EphA2-SE homozygous deletion; GV230 SE^−/−^: Cells transfected with empty vector GV230; GV230-EphA2-SE^−/−^: Cell lines overexpress EphA2.

Many oncogenes regulated by super-enhancers are expressed in tumors at abnormally high levels. In addition, super-enhancers are thought to be involved in biological processes, such as the proliferation and migration of tumor cells as previously described^25,40,41^. RNA-seq data analysis showed that EphA2-SE participates in the growth and metastasis of three tumor cell types. We first explored the effects of EphA2-SE deletion on cell growth using the MTT assay. EphA2 is the target gene of EphA2-SE, and EphA2-SE functions through its target gene. Therefore, we overexpressed EphA2 in HeLa, HCT-116 and MCF-7 cells, and western blotting results showed that EphA2 protein was overexpressed successfully (Fig. 4a). As shown in Fig. 4b, EphA2-SE deletion significantly inhibited cell growth at 72 and 96 h. Overexpression of EphA2 rescued the slower proliferation caused by EphA2-SE deletion. EphA2-SE knockout led to G1/S phase or G2/M phase cell cycle arrest, which is consistent with the result of cell proliferation (Fig. 4c and Fig. S4a). Previous studies have shown that EphA2 is associated with p-AKT, and TCF7L2 is a key transcription factor downstream of the WNT/β-catenin signaling pathway, so we detected PI3K/AKT and WNT/β-catenin. The phosphorylation of AKT protein was decreased in HeLa and MCF-7 cells, but increased in HCT-116 cell. β-catenin and TCF7L2 only decreased in HCT-116 cell. Cell cycle regulation proteins cyclin-E1 showed reduced expression in the SE^−/−^ group cells, resulting in decreased cell proliferation (Fig. 4d). Therefore, EphA2-SE activates different signaling pathways in different tumor cells.

Furthermore, we performed a clonogenic assay to confirm the effects of EphA2-SE on proliferation. The results suggested that the efficiency of colony formation dramatically decreased in EphA2-SE deletion cell lines and rescued by EphA2 overexpression (Fig. 4e and Fig. S4b). Then, we assessed the effect of EphA2-SE on cell mobility. The wound healing assay results showed that cells displayed lower migratory speed in EphA2-SE-deletion cells than in control cells and overexpression of EphA2 displayed faster migratory speed (Fig. 4f and Fig. S4c). We next used transwell to investigate whether EphA2 affects the ability of cancer cells to migrate and invade. EphA2-SE deletion significantly inhibited cell migration and invasion and EphA2 overexpression promotes cell migration and invasion in HeLa, HCT-116 and MCF-7 cells (Fig. 4g and Fig. S4d). These results indicate that the loss of EphA2-SE leads to the decrease of EphA2 expression and significantly inhibited the proliferation, migration and invasion of HeLa, HCT-116 and MCF-7 cancer cells in vitro.

### EphA2-SE deletion suppressed tumorigenesis and tumor proliferation in vivo

**Fig. 5:**
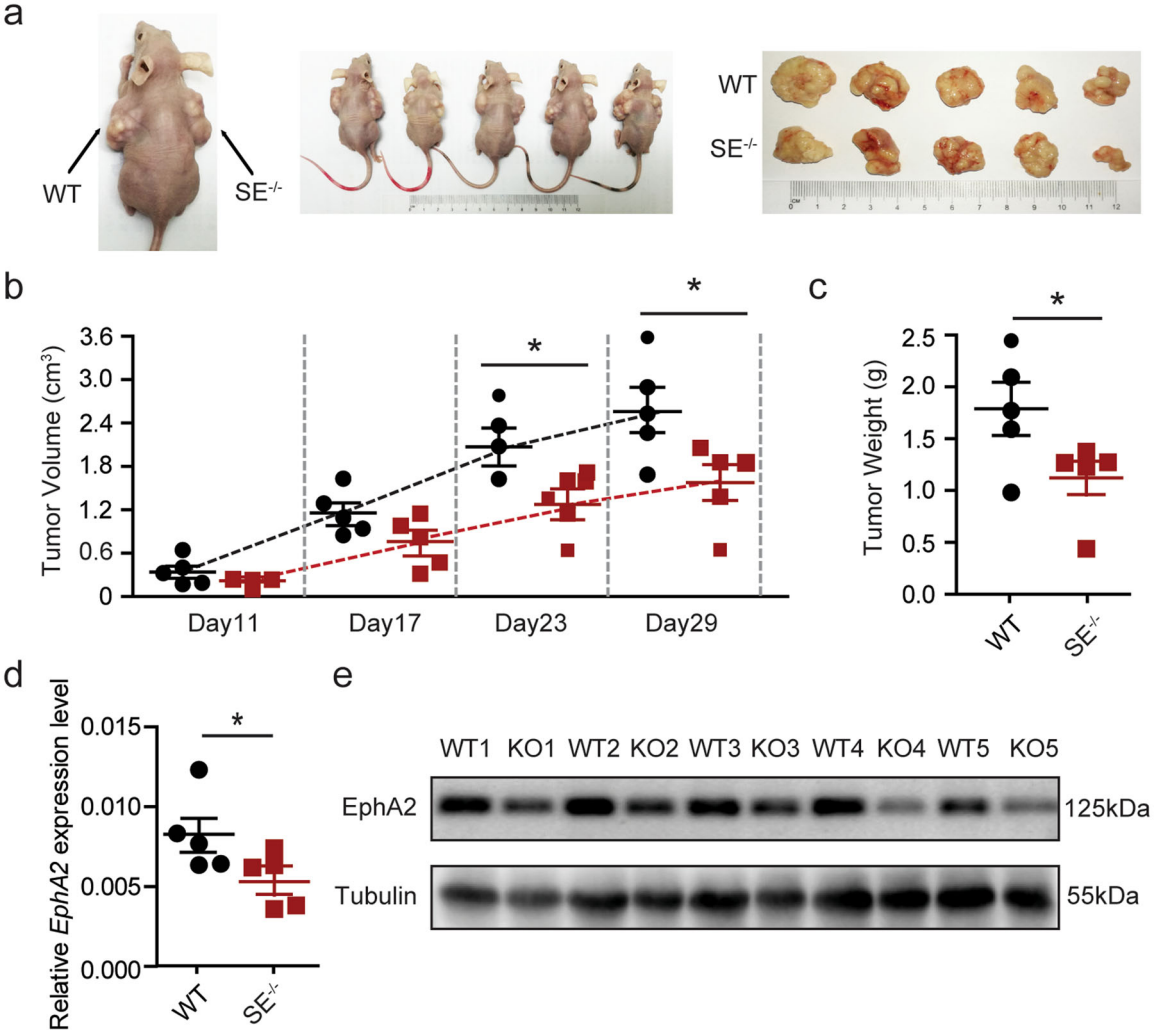
EphA2-SE deletion inhibited tumorigenesis in vivo. **a** Homozygous HeLa clone with EphA2-SE deletion was injected into flanks of nude mice and the representative mice were presented. **b** Tumor growth curve after the injection of EphA2-SE-deficient HeLa cells and control cells into nude mice (*n* = 5). **c** Tumors weight was measured. **d**, **e** The expression of EphA2 in tumors was detected by qRT-PCR and western blotting. Error bars, mean ± SD. *p* values were calculated using *t*-test. *p < 0.05.

To further explore the effects of EphA2-SE on tumorigenesis in vivo, EphA2-SE deletion homozygous HeLa cells and control cells were injected into the flanks of nude mice. The diameters of the tumors were measured every 6 days from the 11th day after cell transplantation. Compared with tumors formed by injection of control cells, tumors formed by EphA2 knockout cells are much smaller in size and weight (Fig. 5a–c). We performed qRT-PCR and western blotting to examine the effect of EphA2-SE on the expression of its target gene. As shown in Fig. 5d-e, the knockout of EphA2-SE dramatically downregulated EphA2 expression. Collectively, these findings indicate that EphA2-SE deletion inhibited the proliferation and growth of HeLa cells, possibly by regulating EphA2 expression in vitro and in vivo.

## Discussion

In recent years, the rapid development of genome-wide sequencing technology has opened up new avenues in the research on super-enhancers. Super-enhancers were first termed as such by Chen et al. in 2004^42^. However, Young and colleagues redefined the super-enhancer in 2013, first clarified the identification method of the super-enhancer, and characterized the function of the super-enhancer in regulating cell identity and disease^2^. Recently, genome-wide association analysis (GWAS) has shown that super-enhancers are associated with many diseases. For example, the coronary artery disease-related GWAS data showed that super-enhancers play a key role in coronary artery disease metabolism through cAMP and ErbB signaling pathways^43^. A super-enhancer in the first intron of LMO1 affects neuroblastoma susceptibility by regulating LOM1 expression^44^. The current research mainly focused on lineage-specific super-enhancers. pDC-specific super-enhancer of *RUNX2* induces high expression of MYC in leukemia cells, and promotes the survival and proliferation of BPDCN cells^40^. A CD47-associated super-enhancer is associated with pro-inflammatory signaling in MCF-7 cell^45^. Only a small number of super-enhancers have been studied for their functional role in a variety of tumors, such as KLF6-SE^25,26^ and MYC-SE^27–30,46^. Disruption of KLF6-SE has been shown to inhibit the growth of liver cancer cells^26^. Syafruddin et al. found a super-enhancer upstream of *KLF6* in kidney cancer, which regulates lipid homeostasis in clear cell renal cell carcinoma (ccRCC) by driving the expression of KLF6^25^. Abnormal DNA non-coding regions will affect the normal expression of genes and play an important role in the occurrence and development of diseases. The research results of Young et al.^4,47,48^ also show that super-enhancers can accelerate the occurrence and development of cancer, and upregulate the expression of genes that promote cancer survival. Currently, pharmaceutical companies are also actively exploring small molecule drugs that can specifically target non-coding regulatory segments of DNA and affect oncogene expression. For example, Syros has developed the main drug candidate SY-5609, which is an inhibitor of cyclin-dependent kinase 7 (CDK7). CDK7 is an important part of enhancers and controls the expression of many oncogenic TFs. Therefore, when we find that a super-enhancer promotes tumor progression in a variety of tumor cells, small molecule drugs targeting the super-enhancer may have a significant therapeutic effect on a variety of tumors. In this study, we identified EphA2-SE in A549, HeLa, MCF-7, HCT-116, Panc-1, T24, MKN45, BT16, A498 and K562 cells.

Many super-enhancers have been confirmed by experiments, such as KLF6-SE^25^, QKI-SE^49^, SOX2-SE^7^ and MYC–SE^39^, which can be searched in super-enhancer databases. In kidney cancer, KLF6-SE promotes the expression of KLF6 and then activates PDGFB to promote lipid metabolism, leading to the growth of ccRCC^25^. YY1/p65/p300 complex binding to the super-enhancer and promoter regions of QKI increases QKI expression to promote the malignancy of HCC^49^. The absence of SOX2-SE in mice affects the expression of many key pluripotency genes^7^. In the lung cancer cell line A549, MYC-SE activated the occurrence of lung cancer cells^39^. In our study, we discovered a super-enhancer at about 15 kb upstream of EphA2 that is present in various tumor cells, including A549, HeLa, MCF-7, HCT-116, Panc-1, T24, MKN45, BT16, A498 and K562 cells from super-enhancer databases. Previous studies have shown that super-enhancers are generally rich in active histone modifications as well as active TFs, such as H3K27ac, Pol2, and DNase I^2^. ChIP experiments and dual-fluorescence analysis indicated that EphA2-SE is a super-enhancer with three component enhancers. The GeneHancer and 4DGenome databases indicate that EphA2-SE interacts with the promoter regions of two genes, EphA2 and ARHGEF19. In HeLa, HCT-116 and MCF-7 cell lines, the expression level of the target gene EphA2 was significantly downregulated after EphA2-SE deletion. However, the expression of ARHGEF19 was only downregulated in MCF-7 cell line with no change or even slight upregulation in HeLa and HCT-116 cells. Therefore, we focus on the EphA2 gene, which is downregulated in all three cells in this study. RNA-seq analysis showed that EphA2-SE is associated with cell growth and migration. Among the common differential genes of the three cell lines, many genes are related to tumor progression. As a target of Mir-19a, THBS1 inhibits the survival, migration, and invasion of colorectal cancer cells^50^. NOV knockdown leads to increased proliferation and invasion of RKO cells^51^. The expression of RGS2 in breast cancer is lower than that in normal tissues, and overexpression of RGS2 inhibits MCF-7 cell proliferation^52^. WNT7B is highly expressed in highly active Wnt/β-catenin pancreatic cancer^53^. Nrp2 blockade reduces tumor lymph angiogenesis and VEGFC-induced lymphatic endothelial cell migration^54^. *EphA2* was also abnormally highly expressed in a variety of tumors and found to be associated with tumor cell proliferation and migration. In gastric cancer cells, EphA2 interacts with YAP to phosphorylate it and promote tumor growth and drug resistance by increasing the stability of the YAP protein^55^. In Ewing sarcoma, p-EphA2S897 silencing significantly inhibits cell migration in vitro and in vivo^56^. EphA2 is highly expressed in colorectal cancer cells and causes high invasiveness and metastasis of cells, and poor prognosis^57^. Our results reveal that EphA2-SE deletion can inhibit tumor cell growth and tumor progression by directly modifying the expression of the adjacent EphA2 gene.

**Fig. 6:**
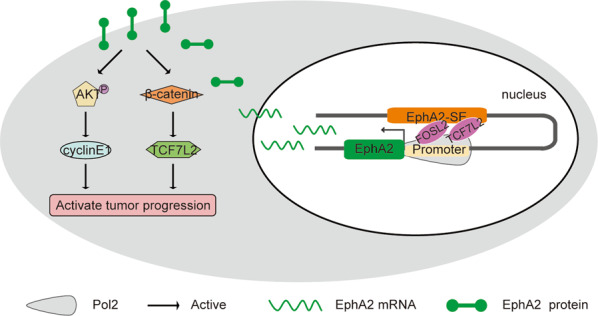
Proposed model of transcriptional regulation of EphA2-SE in HeLa, HCT-116 and MCF-7 cells. EphA2-SE interacts with the EphA2 gene promoter and promotes transcription by recruiting FOSL2 and TCF7L2, resulting in abnormally high expressionof EphA2, which activates signal pathways to promote tumor progression.

In general, super-enhancers promote the expression of their target genes by recruiting a large number of TFs. The transcription factor *YY1* binds to the super-enhanced promoter region of QKI and activates QKI expression, thereby promoting the development of liver cancer^49^. In hepatocellular carcinoma, high expression of the key oncogene HCCL5 is driven by ZEB1 through a super-enhancer^58^. Dual-luciferase reporter assay results suggest that the E1 component enhancer has the strongest activity. Analysis of JASPAR and ChIP-seq data showed that the E1 component enhancer contained FOSL2 and TCF7L2 binding sites. FOSL2 is a member of the AP-1 transcription factor family and is associated with the development of a variety of cancers. In colorectal cancer, silencing of FOSL2 significantly reduces cell migration and has no significant effect on cell proliferation^59^. In hepatocellular carcinoma, FOSL2 is a target gene of MiR-133a, which promotes the proliferation and metastasis of liver cancer cells through the TGF-β /Smad3 signaling pathway^60^. TCF7L2 protein is a key transcriptional effector of the Wnt/β-catenin signaling pathway and mediates resistance of colorectal cancer to chemoradiation^61^. As a transcription factor, TCF7L2 acts synergistically with EGR1 to promote the expression of LCN2 through the ERK pathway, thereby inducing the migration and invasion of esophageal squamous cell carcinoma^62^. After knocking down *FOSL2* and *TCF7L2* using siRNA, EphA2 expression was found to be significantly downregulated. E1 enhancer activity was significantly decreased after the binding sites were deletion. siRNA-mediated downregulation of FOSL2 and TCF7L2 also led to a decrease in E1 activity. After knocking out E1-E3 component enhancers by CRISPR/Cas9 in three cells, the expression of target gene EphA2 was drastically downregulated, and the difference was not large. It may be related to the internal chromatin structure, destroying any component enhancer will break the loop between EphA2-SE and EphA2. Each component enhancer is essential to stabilize chromatin conformation. The E1 component enhancer is only superior to the other two component enhancers in recruiting TFs to promote the activity of target genes, but has the same effect on the spatial conformation of chromatin. MTT and cell cycle assays demonstrated that deletion of EphA2-SE suppressed cancer cell proliferation by blocking PI3K/AKT and WNT/β-catenin signaling pathway. In vivo, deletion of EphA2-SE inhibited the growth of HeLa cells; additionally, the expression of EphA2 expression is downregulated. These results, taken together, indicate that FOSL2 and TCF7L2 bind to different positions of the E1 component enhancer to jointly regulate the expression of *EphA2*, thereby inducing cell proliferation and invasion in HeLa, HCT-116 and MCF-7 cells (Fig. 6).

In summary, a new EphA2-associated super-enhancer was identified in ten cancer cell lines in this study. Results suggested that EphA2-SE recruits TCF7L2 and FOSL2 through the core component E1 to directly target EphA2 and promote tumor progression in cancer cells. Xenograft mouse models and cell experiments in vitro showed that the loss of EphA2-SE attenuates tumor progression. Therefore, EphA2-SE represents a promising potential target for the treatment of various tumors.

## Supplementary information

Supplementary Figure S1

Supplementary Figure S2

Supplementary Figure S3

Supplementary Figure S4

Supplementary Figure Legends

Supplementary Table 1

Supplementary Table 2

Supplementary Materials
