# Supplementary figures and images for: EphA2 super-enhancer promotes tumor progression by recruiting FOSL2 and TCF7L2 to activate the target gene EphA2
Source: Cell Death Dis. 2021 Mar 12;12(3):264. doi: 10.1038/s41419-021-03538-6 (PMC7955082; doi:10.1038/s41419-021-03538-6)

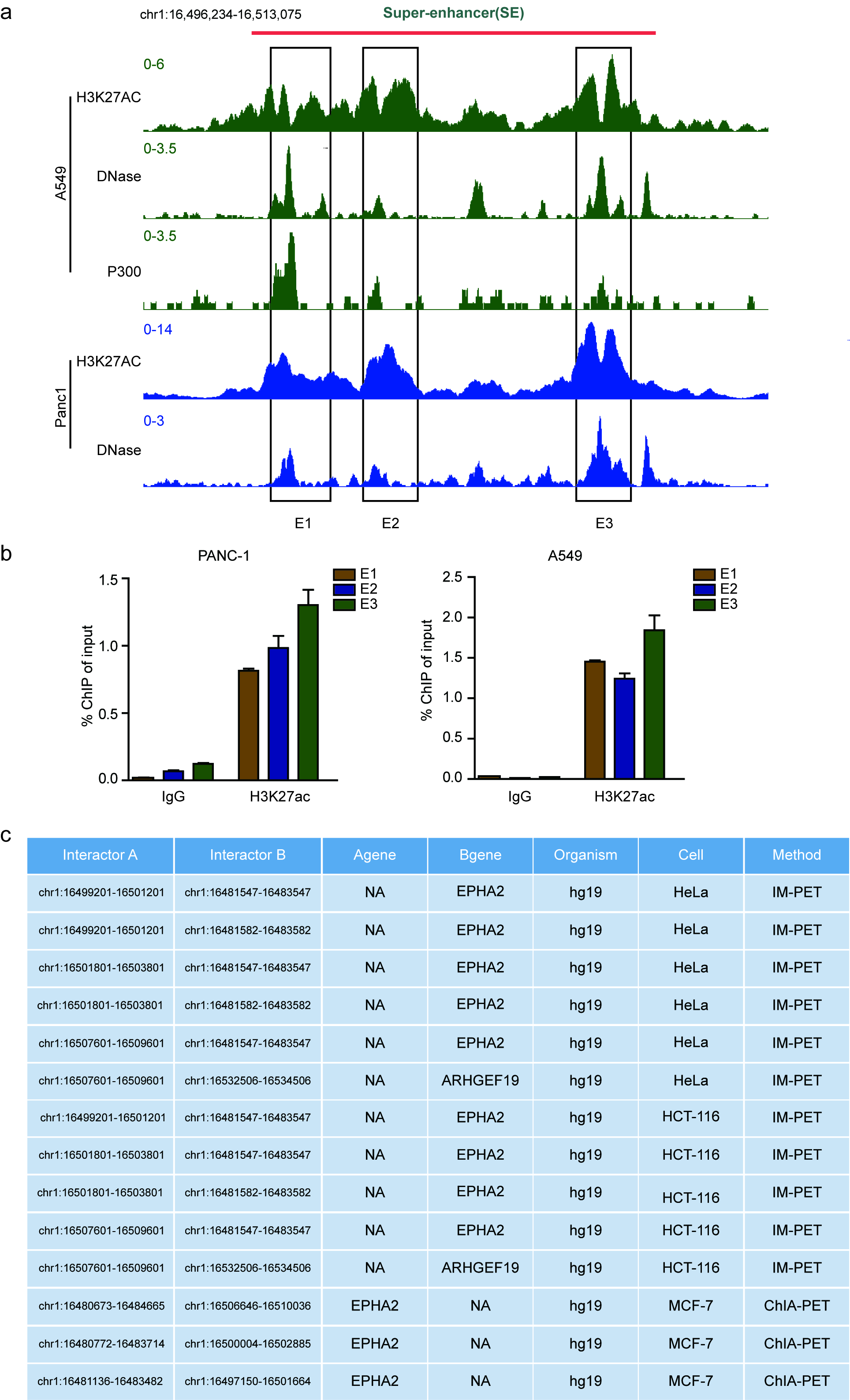

Supplement: Supplementary file 1 — Supplementary Figure S1 [file 41419_2021_3538_MOESM1_ESM.tif]

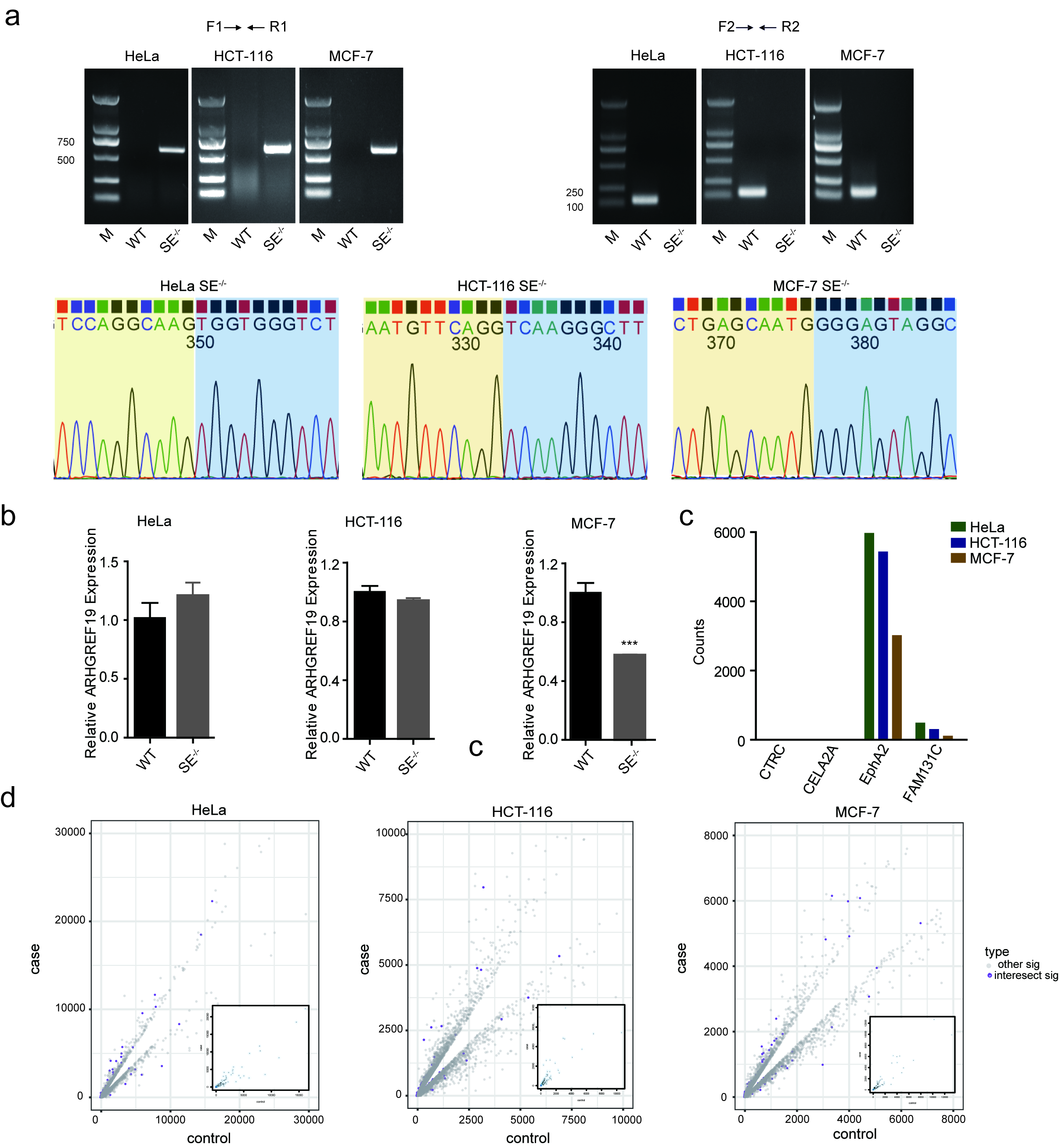

Supplement: Supplementary file 2 — Supplementary Figure S2 [file 41419_2021_3538_MOESM2_ESM.tif]

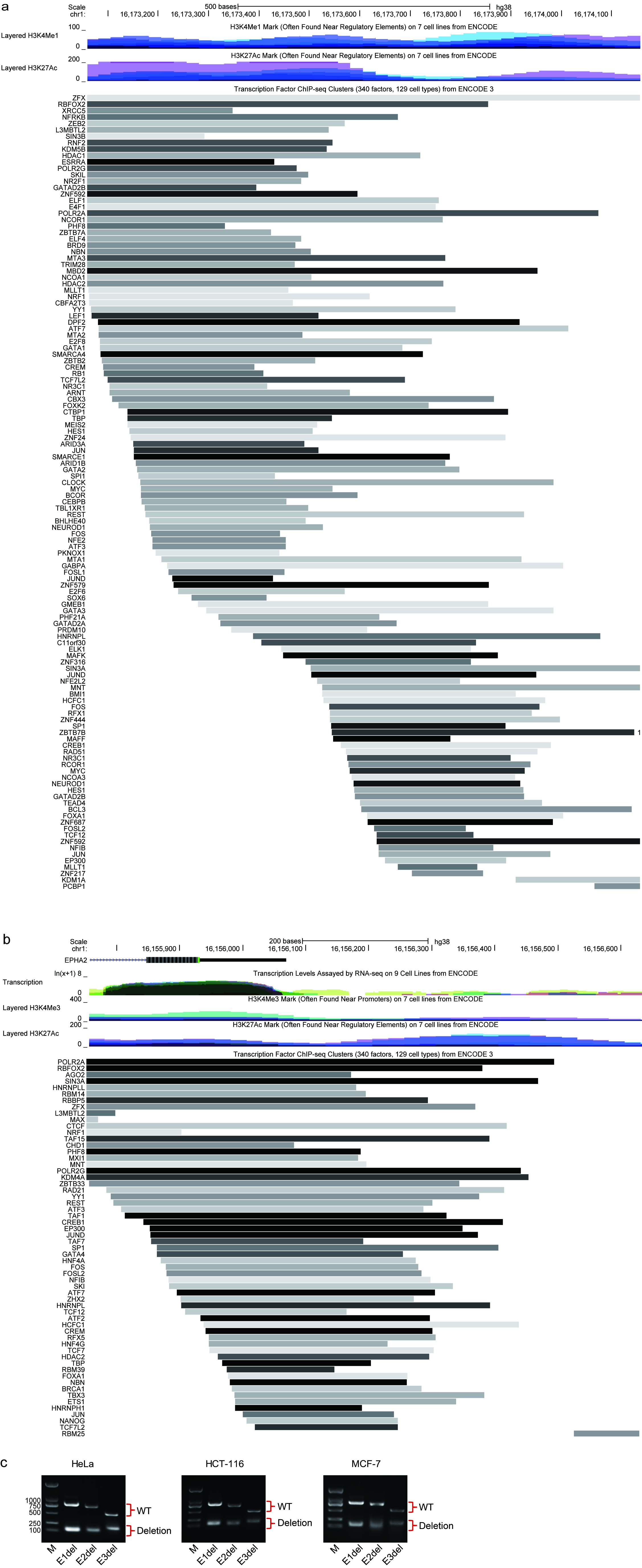

Supplement: Supplementary file 3 — Supplementary Figure S3 [file 41419_2021_3538_MOESM3_ESM.tif]

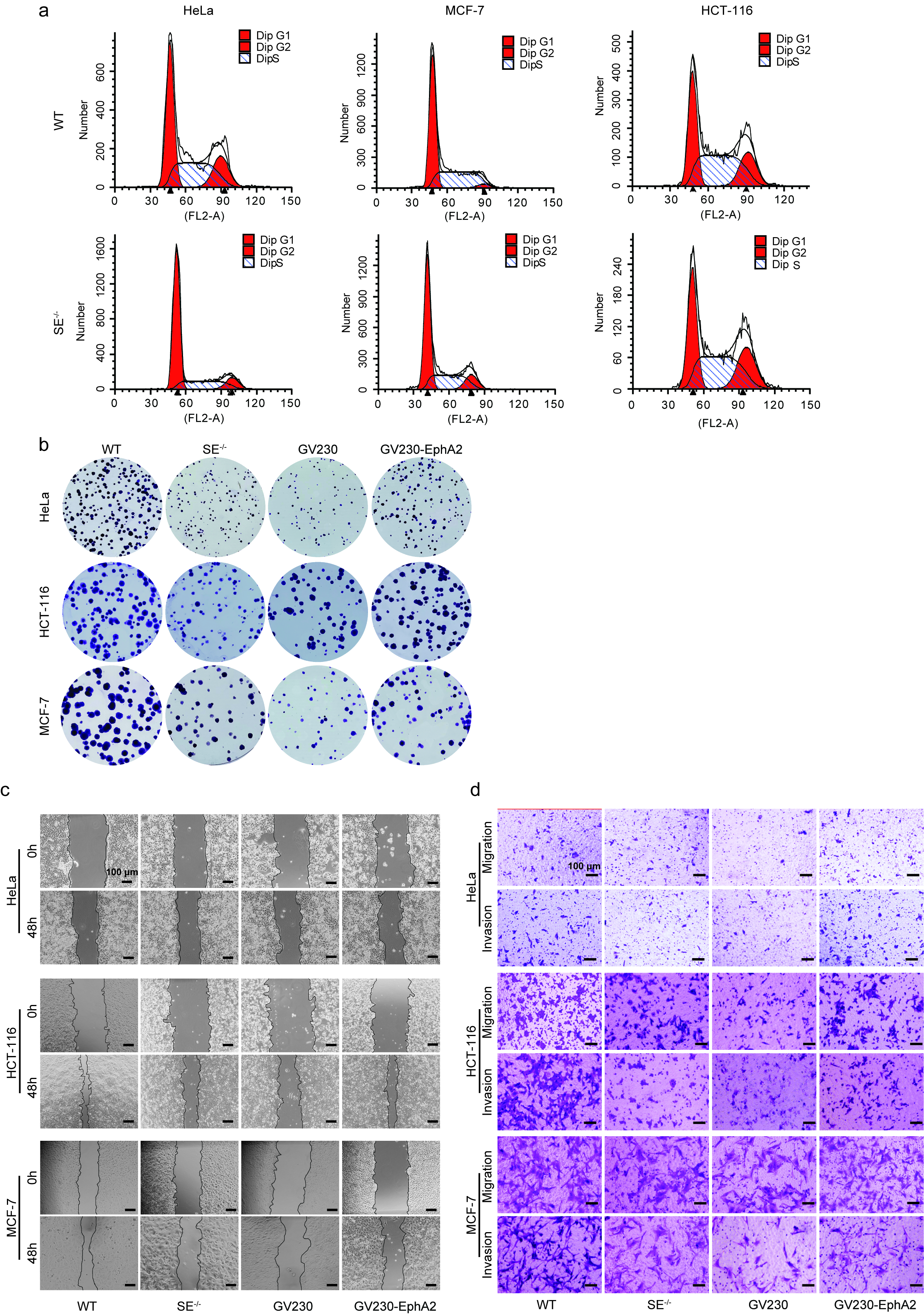

Supplement: Supplementary file 4 — Supplementary Figure S4 [file 41419_2021_3538_MOESM4_ESM.tif]

Map for Modified PX458 Vector


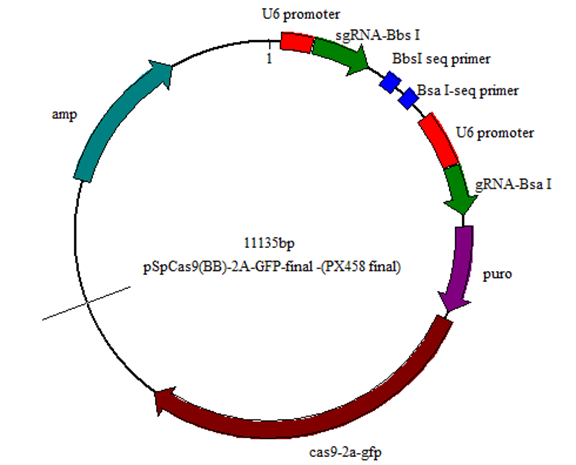

Supplement: Supplementary file 8 — Supplementary Materials [file 41419_2021_3538_MOESM8_ESM.docx]
