# Supplementary material for: EphA2 super-enhancer promotes tumor progression by recruiting FOSL2 and TCF7L2 to activate the target gene EphA2
Source: Cell Death Dis. 2021 Mar 12;12(3):264. doi: 10.1038/s41419-021-03538-6 (PMC7955082; doi:10.1038/s41419-021-03538-6)
Supplement: Supplementary file 5 — Supplementary Figure Legends [file 41419_2021_3538_MOESM5_ESM.docx]

**Supplementary Figure Legends**

**Supplementary Figure S1.** (a) ChIP-seq binding profiles of active modification on the super-enhancers in A549, Panc1 and HepG2 and component enhancer division. (b) ChIP-qPCR analysis of H3K27ac in E1-E3 component enhancers in Panc-1 and A549. (c) The 4DGenome database analyzes the DNA region that interacts with the super enhancer.

**Supplementary Figure S2. Establishment of EphA2-SE-deficien cell lines and RNA-seq analysis.** (a) Top, PCR identified homozygous clones, F1/R1primers were located outside of EphA2-SE, and F2/R2 primers were located inside of EphA2-SE. Bottom, DNA sequencing chromatogram of PCR amplified EphA2-SE deletion clone. The upstream and downstream areas are shown in yellow and blue, respectively. (b) The expression of *ARHGEF19* after EphA2-SE deletion in HeLa, HCT-116 and MCF-7. (c) RNA-seq analyzed the expression of CTRC, CELA2A, EphA2 and FAM131C in HeLa, HCT-116 and MCF-7 cells. (d) Differential genes in HeLa, HCT-116 and MCF-7, respectively.

**Supplementary Figure S3. E1-E3 component enhancers knockout efficiency and E1 master transcription factors recognition.** (a) The EphA2 E1 enhancer region / (b) the promoter region display the active histone modifications and transcription factor modifications of multiple cells (including GM12878, H1-hESC, HeLa-S3, HepG2, HSMM, HUVEC, K562, NHEK and NHLF cell lines) in the ENCODE project in the UCSC browser. The gray box encloses each cluster of peaks occupied by transcription factors, and the darkness of the box is proportional to the maximum signal intensity observed in any cell type contributing to the cluster. More information can be found on the UCSC genome browser. (c) Gel image showing knockout efficiency of E1-E3 enhancer.

**Supplementary Figure S4. Deletion of EphA2-SE inhibited growth and migration of tumor cells *in vitro.*** (a) Effect of EphA2-SE knockout on the cell cycle of HeLa, HCT-116 and MCF-7. (b) EphA2-SE deletion inhibited cell colony formation and EphA2 overexpression rescued the inhibition. (c) Wound healing was used to detect cell migration. (d) Transwell analyzed the invasion and migration.
