# Supplementary material for: EphA2 super-enhancer promotes tumor progression by recruiting FOSL2 and TCF7L2 to activate the target gene EphA2
Source: Cell Death Dis. 2021 Mar 12;12(3):264. doi: 10.1038/s41419-021-03538-6 (PMC7955082; doi:10.1038/s41419-021-03538-6)
Supplement: Supplementary file 6 — Supplementary Table 1 [file 41419_2021_3538_MOESM6_ESM.docx]

**Table1 Primer Sequences**

| **Primer name** | **Primer sequence** |  |
| --- | --- | --- |
| ChIP F1 | TGAAGCCCAGAGAGGCAGTT | ChIP Primers |
| ChIP R1 | AGTGAGGCTCTGGGTGGATG |  |
| ChIP F2 | GAGGGTTAGGGGCAAAGCCA |  |
| ChIP R2 | AAGACGCAGGGACCTGAGAAG |  |
| ChIP F3 | CAGGCTCTGTCCAGATACGG |  |
| ChIP R3 | GGGTGCTCAATAAATGCTTGAGG |  |
| sgRNA1 | TAATAACACGGGCTTGCC | Knockout targets and amplification primers for identifying homozygous clones |
| sgRNA2 | AGTAGGCCTAGGTCTGG |  |
| sgRNA-E1-1 | GAGCCGGCCTGGCAGAGGAG |  |
| sgRNA-E1-2 | GTGACTGGGGGGCAGGTGGG |  |
| sgRNA-E2-1 | TGCTAAAAGGCTCCCGTAGT |  |
| sgRNA-E2-2 | GTACCAGACTTCCTCCCTCG |  |
| sgRNA-E3-1 | TCCAGAGGTTGGTAGGACAT |  |
| sgRNA-E3-2 | CCGGCTGGACCCAGTGCTCG |  |
| External primer F1 | CCGAGTAGTTTGGACCATAG |  |
| External primer R1 | CAAGTTCTTGACTGAATAAGGG |  |
| Internal primer F1 | CAAAGCCAAAGAAATGCC |  |
| Internal primer F2 | GAATGGGTGGATGGATGA |  |
| E1-F | AAACTGAGGCCCAGAGCA |  |
| E1-R | GGCTGGTACAGATTTCAAAGCC |  |
| E2-F | TCATGGTGGCTGGTTGCT |  |
| E2-R | TGGAGCATTTGGGTCCTG |  |
| E3-F | CTGTTCCCTGAAGAGCAA |  |
| E3-R | TCCCGTATCTGGACAGAG |  |
| Componentenhancer F1 | TGGGCGGGGCCAGATCTCGAGCCGAGGTCACTTGAAGGGTATC | Component enhancer primers for construction of dual luciferase reporter vector |
| Componentenhancer R1 | CTGGCCTAACTGGCCGGTACCGGCTTCAGCCCCATTCTAGG |  |
| Componentenhancer F2 | TGGGCGGGGCCAGATCTCGAGGTCTCTCCAGAGCAGGAACGGA |  |
| Componentenhancer R2 | CTGGCCTAACTGGCCGGTACCCACTGAGGACAGTCCCTCTCACT |  |
| Componentenhancer F3 | TGGGCGGGGCCAGATCTCGAGACTTCCCCATCCCAGCTAAC |  |
| Componentenhancer R3 | CTGGCCTAACTGGCCGGTACCCCTTGAGACTCCAGGCTTCA |  |
| A segment -F | CCGCTCGAGAGGAGTTCACGGGGCCTCTT |  |
| A segment -R | CGGGGTACCGACAAAGGCTGGGAGTAGTG |  |
| FOSL2-De-F1 | CGCCTGGGGATAGGGCTTGCTTTAGG | E1 enhancer transcription factor binding site deletion primers |
| FOSL2- De -R1 | GGCTGGGCAGCTCTGGCACTCCCAG |  |
| FOSL2- De -F2 | TTTTATTATATGAGATAAGGCGGCTGG |  |
| FOSL2- De -R2 | TCCTCAGCCTCTGTCTTTCATAAAG |  |
| TCF7L2- De -F1 | GAGGCTGAGGAGAGTGAGTCAGTTTTA |  |
| TCF7L2- De -R1 | AGGGGGAACCTGAGGTGTGGTTAGTTCT |  |
| TCF7L2- De -F2 | GTACCAGCCATGTGACTTGAACAAGGCC |  |
| TCF7L2- De -R2 | CATGACCTCACTCCATCCCCCTG |  |
| si-TCF7L2-sense1 | CAUUCAUGUUGUAUAUGAAGG | siRNA sequences |
| si-TCF7L2-antisense1 | UUCAUAUACAACAUGAAUGCA |  |
| si-TCF7L2-sense2 | GGCUCACUCCAUAGUUCAAAG |  |
| si-TCF7L2-antisense2 | UUGAACUAUGGAGUGAGCCGA |  |
| si-FOSL2-sense1 | GGCCCAGUGUGCAAGAUUAGC |  |
| si-FOSL2-antisense1 | UAAUCUUGCACACUGGGCCGU |  |
| si-FOSL2-sense2 | CGAACCUCGUCUUCACCUAUC |  |
| si-FOSL2-antisense2 | UAGGUGAAGACGAGGUUCGAG |  |
| EphA2-F | TGCCAGTGTCAGCATCAACCAG | qRT-PCR Primers |
| EphA2-R | AGTCTCCCTTCTTGCGGTAAGTG |  |
| ARHGEF19-F | TCGCTGTGGCAGGATATCCC |  |
| ARHGEF19-R | CGACAGGCTGTGGATGTAGGAG |  |
| NRP2-R | GGATGATCTCCATCTTGGGTTTGG |  |
| RGS2-F | GGAAGCCCAAAACCGGCAAA |  |
| RGS2-R | AAGCCCTGAATGCAGCAAGAC |  |
| NOV-F | AGATCTGGAGCCATGCGA |  |
| NOV-R | GTAGATGACCCCATCGAACAC |  |
| THBS1-F | AAGGACAACTGCAGACTC |  |
| THBS1-R | CATTTTGGGATGTCCCTTTG |  |
| FOSL2-F | ACATGGCCCTCCCAAGACCT |  |
| FOSL2-R | GCTGCAGCCAGCTTGTTCCT |  |
| TCF7L2-F | AATCGTCCCAGAGTGATG |  |
| TCF7L2-R | GTATTTCGCTTGCTCTTCTC |  |
| RHOB-F | AGAGGGAAAAGAAACCCA |  |
| RHOB-R | ACATAGCTTTAGAGAATGCC |  |
| FOSL2-de sequence 1 | TGCTGACTCAG | Deleted motif sequence |
| FOSL2-de sequence 2 | GAGTGAGTCAG |  |
| TCF7L2-de sequence 1 | TCF7L2-de sequence 1 |  |
| TCF7L2-de sequence 2 | AGGCTTTGAAATCT |  |
