# Supplementary material for: EphA2 super-enhancer promotes tumor progression by recruiting FOSL2 and TCF7L2 to activate the target gene EphA2
Source: Cell Death Dis. 2021 Mar 12;12(3):264. doi: 10.1038/s41419-021-03538-6 (PMC7955082; doi:10.1038/s41419-021-03538-6)
Supplement: Supplementary file 7 — Supplementary Table 2 [file 41419_2021_3538_MOESM7_ESM.docx]

Table 2. ChIP-seq is used to identify and analyze super-enhancers

| Cell Type | Histone Marker / TF | GEO or ENCODE |
| --- | --- | --- |
| HeLa | H3K27ac | GSM2029136 |
| HCT-116 |  | GSM2534278 |
| MCF-7 |  | ENCSR752UOD_1 |
| Panc-1 |  | GSM2466034 |
| A549 |  | GSM2406904 |
| K562 |  | GSM2877103 |
| BT16 |  | GSM1835887 |
| MKN45 |  | GSM2076193 |
| T24  A498 |  | GSM1948906  GSM2723838 |
| 293T |  | GSM2439222 |
| Colon Crypt |  | GSM883685 |
| MCF-10A |  | GSM1874951 |
| Pancreas |  | GSM1606427 |
| Lung |  | GSM646476 |
| Stomach |  | GSM1252312 |
| HeLa | DNase | GSM763533 |
| HCT-116 |  | GSM1224667 |
| MCF-7 |  | GSM736581 |
| Panc-1 |  | GSM736517 |
| A549 |  | GSM736506 |
| HeLa | EP300 | GSM1517093 |
| HCT-116 |  | GSM1240110 |
| MCF-7 |  | GSM1010800 |
| A549 |  | ENCSR792VMN_2 |
